# Baseline results of a living systematic review for COVID-19 clinical trial registrations

**DOI:** 10.12688/wellcomeopenres.15933.1

**Published:** 2020-06-02

**Authors:** Brittany J. Maguire, Alistair R.D. McLean, Sumayyah Rashan, Emilia Sitsofe Antonio, Jayshree Bagaria, Zineb Bentounsi, Matthew Brack, Fiona Caldwell, Verena Ilona Carrara, Barbara Wanjiru Citarella, Prabin Dahal, Vitalis Fambombi Feteh, Marius H.B. Guérin, Kalynn Kennon, Kathinka Bilton Lahaut, Gerald Jamberi Makuka, Roland Ngu, Sopuruchukwu Obiesie, Caitlin Richmond, Sauman Singh-Phulgenda, Samantha Strudwick, Carina S.B. Tyrrell, Austin Schwinn, David King, Paul N. Newton, Ric N. Price, Laura Merson, Kasia Stepniewska, Philippe J. Guérin

**Affiliations:** 1 Infectious Diseases Data Observatory (IDDO), Oxford, UK; 2 Centre for Tropical Medicine and Global Health, Nuffield Department of Medicine, University of Oxford, Oxford, UK; 3 Nuffield Department of Orthopaedics, Rheumatology and Musculoskeletal Sciences, University of Oxford, Oxford, UK; 4 Shoklo Malaria Research Unit, Mahidol-Oxford University Research Unit, Mahidol University, Mae Sot, Tak, Thailand; 5 Institute of Global Health, Faculty of Medicine, University of Geneva, Geneva, Switzerland; 6 Nuffield Department of Medicine, Big Data Institute, University of Oxford, Oxford, UK; 7 Health and Human Development (2HD) Research Network, Douala, Cameroon; 8 Faculty of Medicine, University of Oslo, Oslo, Norway; 9 Muhimbili University of Health and Allied Sciences, Dar es Salaam, Tanzania; 10 Public Health England, London, UK; 11 MRC Epidemiology Unit, University of Cambridge, Cambridge, UK; 12 Exaptive, Inc., Oklahoma City, Oklahoma, USA; 13 Lao-Oxford-Mahosot Hospital-Wellcome Research Unit (LOMWRU), Microbiology Laboratory, Mahosot Hospital, Vientiane, Lao People's Democratic Republic; 14 Global and Tropical Health Division, Menzies School of Health Research, Charles Darwin University, Darwin, NT, Australia; 15 Mahidol-Oxford Tropical Medicine Research Unit, Faculty of Tropical Medicine, Mahidol University, Bangkok, Thailand; 16 International Severe Acute Respiratory and Emerging Infection Consortium (ISARIC), Oxford, UK

**Keywords:** Living systematic review, COVID-19, SARS2-CoV2, coronavirus, clinical trials, emerging infections

## Abstract

**Background: **Since the coronavirus disease 2019 (COVID-19) outbreak was first reported in December 2019, many independent trials have been planned that aim to answer similar questions. Tools allowing researchers to review studies already underway can facilitate collaboration, cooperation and harmonisation. The Infectious Diseases Data Observatory (IDDO) has undertaken a living systematic review (LSR) to provide an open, accessible and frequently updated resource summarising characteristics of COVID-19 study registrations.

**Methods: **Review of all eligible trial records identified by systematic searches as of 3 April 2020 and initial synthesis of clinical study characteristics were conducted. In partnership with Exaptive, an open access, cloud-based knowledge graph has been created using the results.

**Results: **There were 728 study registrations which met eligibility criteria and were still active. Median (25 ^th^, 75 ^th^ percentile) sample size was 130 (60, 400) for all studies and 134 (70, 300) for RCTs. Eight lower middle and low income countries were represented among the planned recruitment sites. Overall 109 pharmacological interventions or advanced therapy medicinal products covering 23 drug categories were studied. Majority (57%, 62/109) of them were planned only in one study arm, either alone or in combination with other interventions. There were 49 distinct combinations studied with 90% (44/49) of them administered in only one or two study arms. The data and interactive platform are available at https://iddo.cognitive.city/.

**Conclusions:**  Baseline review highlighted that the majority of investigations in the first three months of the outbreak were small studies with unique treatment arms, likely to be unpowered to provide solid evidence.  The continued work of this LSR will allow a more dependable overview of interventions tested, predict the likely strength of evidence generated, allow fast and informative filtering of relevant trials for specific user groups and provide the rapid guidance needed by investigators and funders to avoid duplication of efforts.

## Introduction

The urgent need for immediate solutions to tackle public health emergencies of international concern undermines effective coordination and collaboration between researchers ^1, 2^. Since the coronavirus disease 2019 (COVID-19) outbreak was reported in December 2019, a huge number of clinical studies have been initiated, firstly in China, South Korea, then subsequently in Europe, Japan, North America and Australia. The number of trials recorded in clinical trial registries is proliferating exponentially, with many independent research efforts designed to answer similar questions. Whilst multicentred trials can potentially improve generalisability of study results across widely divergent endemic settings, this requires comparable study designs and comparators. Conversely, independent trials with small sample sizes and underrepresentation of some patient populations limit the acquisition of substantive evidence to assess the effects and applicability of the many diagnostic methods, therapeutic and prophylactic strategies targeted to address the COVID-19 pandemic ^3^. As the research landscape becomes increasingly saturated, better coordination in study design, dosing strategies and pooling data for subsequent metanalyses, are needed to ensure definitive answers can be translated into clinical practice as rapidly as possible. Tools that allow researchers to review the diversity of interventions already underway are critical in facilitating collaboration, harmonisation, partnerships and open access research.

Methods to distil the current research environment can help identify studies likely to be adequately powered to answer research questions, key research gaps that still need to be addressed, as well as duplication of efforts. Furthermore, as results accrue it becomes clear which scientific questions can only be addressed by pooling and standardising individual patient-level data across different trials. Since early 2000 there has been progressive demand from scientific journals and funders for investigators to register trials to monitor health research conducted on humans; over 21 registries are now available ^4– 8^. Although trial registries do not capture all research conducted for a specific theme, they provide an accepted source and proxy of the volume of research either proposed or underway ^9, 10^. To support these efforts and to provide clarity regarding study design, including objective measures of quality and power to generate reliable evidence of the safety, efficacy, and utility of interventions, a living systematic review permitting regular and maintained search updates of registered COVID-19 clinical trials is underway ^11^.

The primary objective of this living systematic review is to provide an open, accessible and frequently updated resource summarising the characteristics of COVID-19 clinical study registrations. Herein, we present the results of the baseline review of all eligible trial records identified by the systematic searches as of 3 April 2020 and initial synthesis of clinical study characteristics.

## Methods

### Protocol

The living systematic review protocol was prospectively designed and published elsewhere ^12^, containing extensive details of the methodology and rationale for use of the living method.

### Eligibility criteria

All clinical trial registrations either being planned, or currently underway to diagnose, treat or prevent COVID-19 were eligible for inclusion in the baseline results of this review. This included patients known to be infected with COVID-19 as well as healthy volunteers, healthcare workers or other patient populations where health related outcomes were assessed in the context of COVID-19. The review was not limited by outcome, language or intervention given the desire to capture all clinical trial registrations planning to or currently evaluating any COVID-19 diagnostic, prevention or treatment modality. Eligibility was not restricted to clinical trials only; no limitations on study design were applied so that all research and observational studies would be incorporated, including retrospective studies. All trial registry records included in the review are hereafter referred to as clinical ‘study’ registrations.

### Information sources and search strategy

Formal searches for clinical trial registrations were conducted as detailed in the protocol publication ^12^ up until 23 March 2020. Due to heavy traffic on the World Health Organization (WHO) International Clinical Trials Registry Platform (ICTRP) which aggregates records from 17 country and regional trial registries ^4^, on 24 March 2020 this central information source was no longer accessible to anyone outside of the WHO. A download of all COVID-19 trials from the ICTRP database was made available to us on 1 April 2020, and the latest export publicly available for download on 3 April which was derived using the search terms *((COVID-19) OR (novel coronavirus) OR (2019-ncov))* is the last search update used for inclusion in the baseline results of this review. The source registry records which supplemented all included studies identified through the WHO ICTRP, were accessed live on the dates of data extraction for each record and not from an archived version.

### Search and update schedule

This article presents the baseline results of the systematic review. Searches for this living systematic review are performed weekly and updates to the review following data extraction of newly identified and included records will be made available via the IDDO COVID-19 Living Systematic Review Website as completed ^13^. Searches and updates will continue for the foreseeable future during 2020. This article will be updated every 6 months. The rationale for the search schedule is provided in the study protocol ^12^.

### Study records, data items and outcomes

The process for study selection and data for all eligible trial records were identified and extracted as per the published protocol in a standardised, pre-piloted REDCap database ^12, 14, 15^. This included automated imports of WHO ICTRP data using Trifacta® Wrangler (Trifacta Inc. USA), blinded screening of trial records for eligibility by at least two reviewers [BM, SR], and data entry of additional manual variables completed by one reviewer with each variable cross-checked for quality control by a second reviewer. No records were excluded at the title level, each record was reviewed in its entirety either as reported in WHO ICTRP or the source registry before assessment of eligibility. All data items including their classifications, assumptions, rules and definitions for extraction are presented in the variable and data dictionaries available as extended data ^13^. These data, along with all of the results data, analysis scripts and supplementary tables and figures have been deposited and are available from an open access repository or from the IDDO COVID-19 Living Systematic Review Website ^13^.

Definitions of the study designs manually assigned to records in this review were as per the Cochrane Consumers & Communication Review Group Study Design Guide ^16^. Interventional studies were categorised as ‘randomised control trials (RCT)’; ‘quasi-randomised trials’; or ‘non-randomised interventional studies’. For observational and epidemiological research one of the following study designs were selected: ‘cohort-study’; ‘case report’; ‘case series’; ‘case-control’; ‘cross-sectional’; or ‘prognostic’. For any other study designs, including health services research, behavioural and social science studies, the category of ‘other’ was attributed. A final study design of ‘diagnostic test accuracy’ was assigned where the primary outcome was assessment of diagnostic methods. The World Bank list of economies (June 2019) was used for income classification of countries ^17^.

The taxonomy of interventions was adapted from the initial categories designed by The CEBM Oxford COVID-19 Evidence Service ^18^ and extended for any new treatments identified. At the highest level, interventions within each study arm were categorised as: “Pharmacological interventions”; “Traditional Chinese Medicine”; “Vaccine prevention”; “Vaccine treatment”; “Advanced therapy medicinal products (ATMP)” [defined as a gene therapy medicinal product; a somatic cell therapy medicinal product; or a tissue engineered product ^19^]”; “Behavioural intervention” [including social science studies], “Placebo”, “No Intervention” [where explicitly reported or zero details provided], “Standard of care ” [where standard of care, routine treatment/therapy, best supportive care, general treatment, conventional treatment, standard treatment or related synonyms were explicitly reported], “Diagnostic intervention”; or “Others ” [including nutritional supplements and enteral feeds; physiotherapy and exercise; physical therapies, such as renal replacement therapy; psychotherapies]. Pharmacological interventions and ATMP categories were further sub-classified in alignment with the CEBM taxonomy (see variable dictionary for further details) ^13, 18^. Without available expertise and a lack of standards in the reporting, further examination of “Traditional Chinese Medicine” interventions was not undertaken.

The baseline data for this analysis were not supplemented by further details from the investigators regarding ambiguous registration details or unreported trial features.

### Synthesis of results

Descriptive statistics were used to present the extracted data. Categorical variables were summarised with proportions and frequencies; continuous variables were summarised with totals, means, quartiles, minimums and maximums. Summary statistics and figures were produced using Stata 14.2 (StataCorp, College Station, TX, USA) and R software (version 3.6.3, The R Foundation for Statistical Computing, Vienna, Austria).

In partnership with Exaptive, an open access, cloud-based knowledge graph has been created using the baseline systematic review results ^20, 21^. Leveraging Exaptive’s virtual collaboration platform, the “ Cognitive City”, built upon a graph-based architecture at its core, uses the REDCap database from the living systematic review to allow stakeholders to query the data generated. Ongoing search updates are scheduled to be incorporated into this open, online, searchable tool as they become available.

### Risk of bias

The living systematic review of registered trials does not address specific clinical questions. Baseline results are limited to the initial data extraction, descriptive analysis of trial characteristics and visualisation of clinical trial registry records. Accordingly, assessment of meta-biases or the strength of the body of evidence represented by included records are not relevant. In a first effort to distil the hierarchy of evidence being generated for COVID-19 interventions, registration details have been used to make a preliminary assessment of the strength and potential bias in relation to the design and intended execution within individual studies using the Oxford Centre for Evidence-Based Medicine 2011 Levels of Evidence ^22^.

## Results

### Study selection

**Figure 1.**
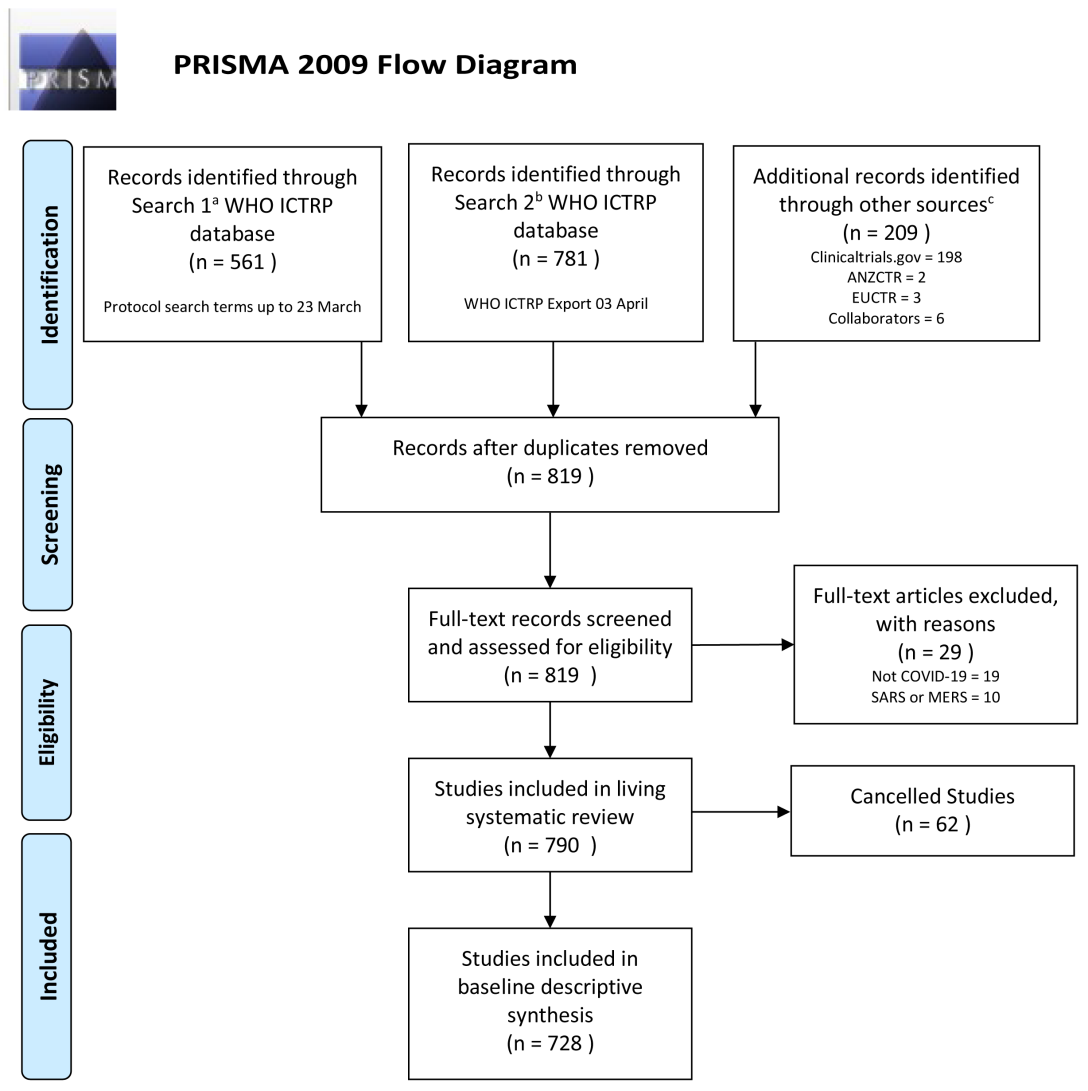
PRISMA Flow Diagram Living Systematic Review Registry Searches for Baseline Results at 3 April 2020. All de-duplicated records screened, assessment of eligibility, list of included records available as supplementary materials, see Data Availability. **a** Per protocol search terms of ((COVID-19) OR (coronav*) OR (*CoV-2) OR (nCoV*)) **b** Search terms as per only publicly available WHO ICTRP registry export of COVID-19 trials, compiled by WHO ICTRP using the terms ((COVID-19) OR (novel coronavirus) OR (2019-ncov)) **c** Other sources last searched as per protocol up to 31 ^st^ March 2020. Abbreviations: WHO ICTRP, World Health Organization International Clinical Trials Registry Platform; ANZCTR, Australia New Zealand Clinical Trials Registry; EUCTR, European Clinical Trials Registry. The latest and previous versions of this figure are available as extended data ^13^.

A total of 819 unique trial registry records were screened and assessed by at least two independent reviewers against the pre-specified eligibility criteria. The PRISMA flow diagram and summary of the literature screening is provided in Figure 1. Of the 819 unique records, 29 records were excluded because they did not include COVID-19 patients, and were not conducted in the context of COVID-19. Of the 790 records that met eligibility, 62 clinical trials were cancelled after registration and therefore excluded from further analysis.

### Study characteristics

**Figure 2.**
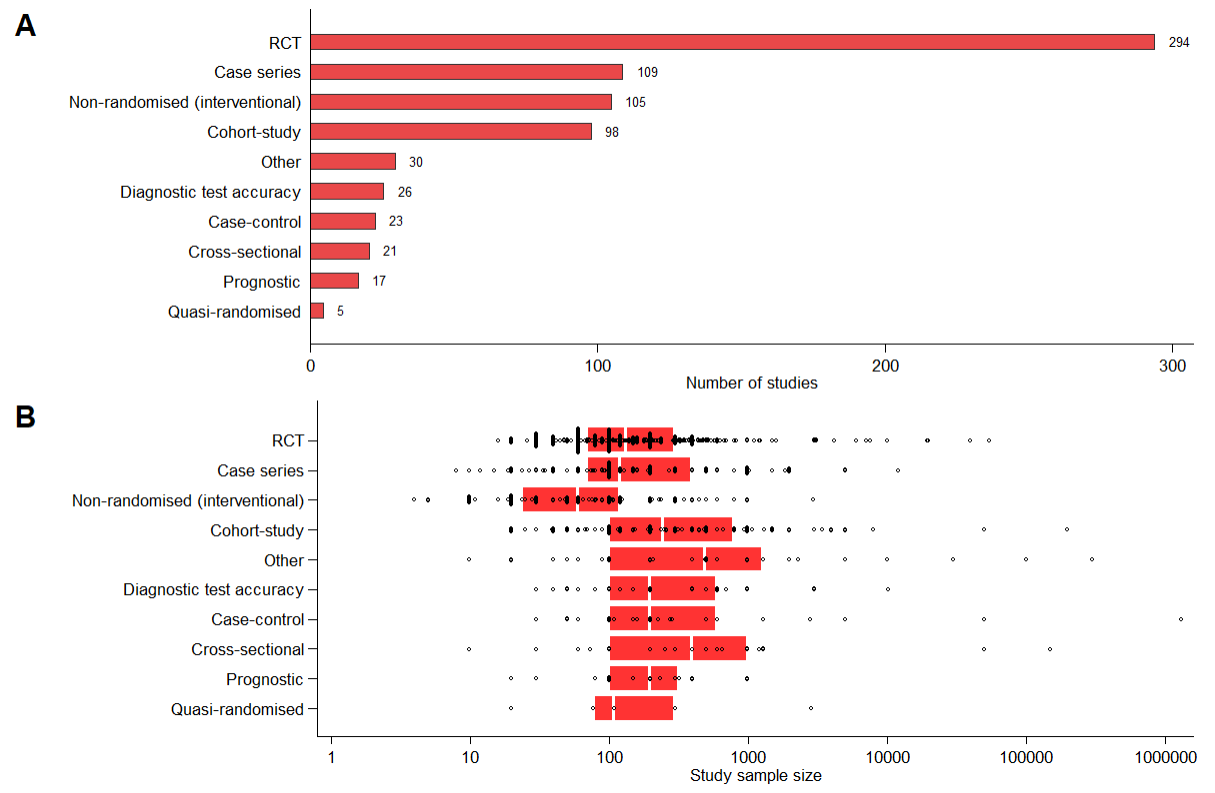
( **A**) Number of studies and ( **B**) planned sample size by design type. Planned sample size was unknown for two non-randomised (interventional) studies and 1 other study. Each black circle denotes one trial; red box denotes 25 ^th^ and 75 ^th^ percentiles; vertical white line indicates the median. The latest and previous versions of this figure are available as extended data ^13^.

The most common registered study design identified was randomised control trial (RCT), with 294 studies identified ( Figure 2A). Other study designs frequently identified were: case series (N=109), non-randomised interventional studies (N=105), and cohort studies (N=98). There were relatively few studies classified as diagnostic test accuracy (N=26), case-control (N=23), cross-sectional (N=21), prognostic (N=17) or quasi-randomised (N=5). In total 29 (4%) studies did not contain sufficient information in the registry to determine the study design. A total of 435 studies involved a treatment intervention, 56 involved a diagnostic intervention and 38 involved a preventative intervention.

Most studies identified had proposed relatively small sample sizes; the median (25 ^th^, 75 ^th^ percentile) sample size for all studies was 130 (60, 400). Reported sample sizes were generally low across all study designs, with median (25 ^th^, 75 ^th^ percentile) sample sizes of 134 (70, 300) for RCTs and 60 (24, 120) for non-randomised interventional studies ( Figure 2B). Cohort studies tended to have larger sample sizes with median (25 ^th^, 75 ^th^ percentile) 245 (100, 800). Of the 728 included studies, 207 plan to assess a pharmacological intervention, 132 plan to assess a traditional Chinese medicine, 48 plan to assess an advanced therapy medicinal product; 39 plan to assess a diagnostic intervention and 28 trials plan to assess a behavioural intervention. There were six studies identified in this search planning to assess a preventative vaccine and three studies planning to assess a vaccine treatment. Reviewers considered 317 (44%) of studies to be level 2 on the Oxford CEBM levels of evidence scale, 188 (26%) level 3; 216 (30%) level 4 and 7 (1%) level 5. At the time of data extraction, 54% (392) of identified studies were registered as currently recruiting participants.

**Figure 3.**
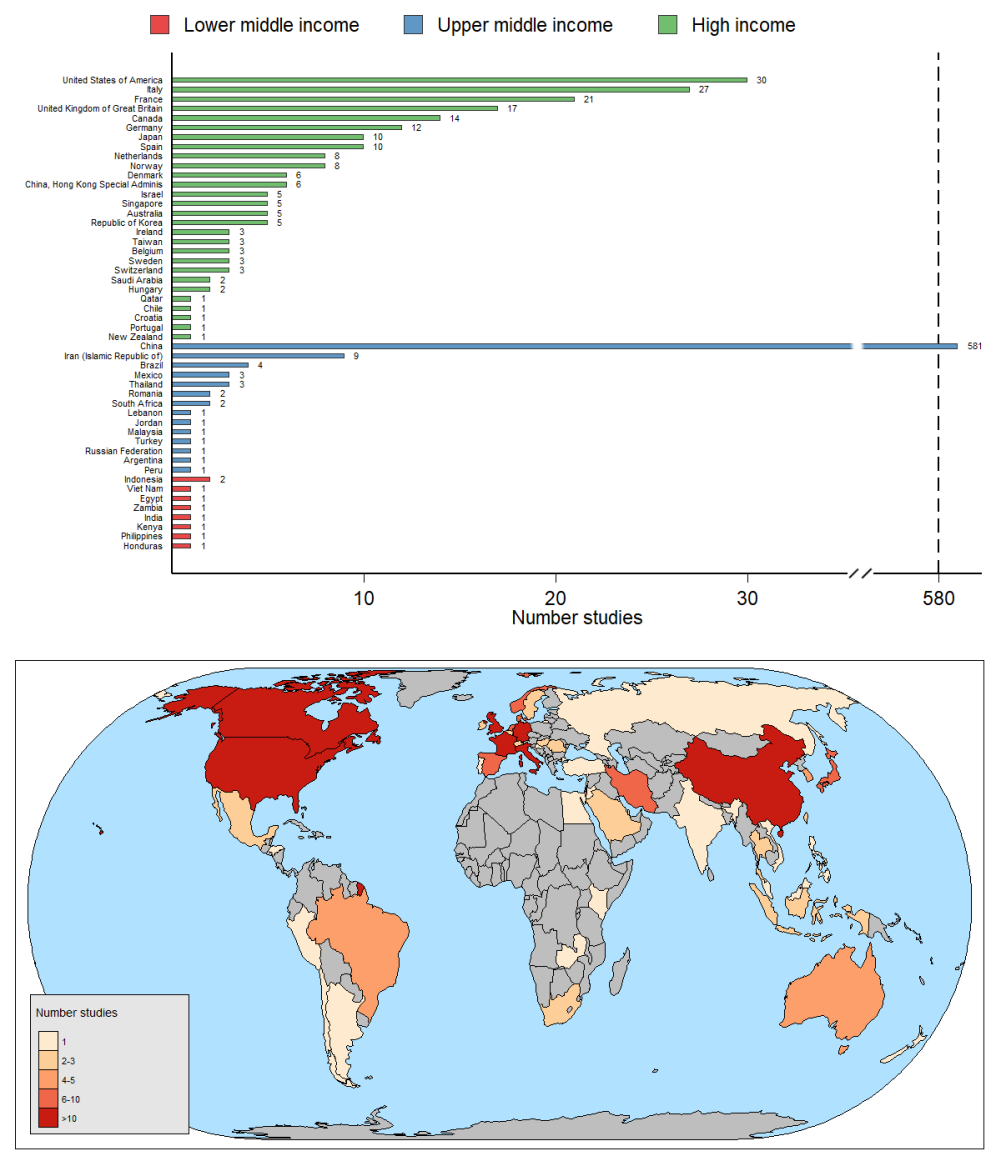
Number of registered studies planning to recruit participants in each country. Note the break in the x axis between 40 and 580. The latest and previous versions of this figure are available as extended data ^13^.

***Planned location of studies***. The majority of registered studies (74%, 527/728) involved a single study site with only 25% (185/728) of studies planning to recruit participants from multiple sites in the same country and 2% (16/728) of studies planning to recruit participants from multiple countries. The vast majority of registered studies (80%, 581/728) planned to recruit participants from China ( Figure 3). Only four studies included in the review included sites located in low income or lower middle income countries, with only 17% (8/47) of lower middle income countries represented in the studies and 0% (0/31) of low income countries. Conversely, 35% (28/80) of high income countries and 30% (14/47) of upper middle-income countries were represented by proposed recruitment sites.

***Eligible populations***. Discerning whether at-risk populations were eligible for inclusion in the study was not always possible from the registry information (Supplementary Table 1 – see extended data ^13^). Of the risk groups investigated, 365 (50%) studies explicitly reported planned exclusion of pregnant women, 9 (1.2%) studies planned inclusion of pregnant women, and for the remaining 354 (49%) studies there was insufficient information to classify the study. A total of 110 (15%) studies planned to include children (<18 years), in 491 (67%) studies children were not eligible for inclusion and in 127 (17%) studies there was insufficient information on age eligibility. Most study registrations contained insufficient information to classify the eligibility of patients with: diabetes mellitus (94% unknown); HIV (84% unknown); immunocompromised condition (82%); chronic lung disease or moderate to severe asthma (82%); hypertension (96%); serious heart conditions (77%); severe obesity (99%).

***Study arms and planned sample size by type of Intervention***. In total there were 389 study arms assessing a pharmacological intervention or ATMP, with the majority coming from RCTs (297, 76%). Among RCTs, quasi-randomised, cohort/non-randomised, case series and case-control studies (385 arms), the most common types of pharmacological interventions were antivirals (164 arms, 43%), antimalarials (76 arms, 20%), ATMPs (60 arms, 16%), and monoclonal antibodies (31 arms, 8%). For full list see Table 1.

**Table 1.** Summary of pharmacological and ATMP interventions studied in case series, case-control, cohort/non-randomised, quasi-randomised and randomised studies. The latest and previous versions of this table are available as extended data ^13^.

Drug Name	Number of studies	Number of arms	Number of arms with Sample Size	Sample Size Total	Median	IQR	Range	Number of arms with combination therapy
**Case Series**								
**ALL**	**4**	**4**	**4**	**110**	**25**	**20 - 35**	**20 - 40**	
**ATMP**	**2**	**2**	**2**	**70**	**35**	**30 - 40**	**30 - 40**	
Mesenchymal Stem Cells	1	1	1	30	30			
Aerosol inhalation of vMIP: viral macrophage inflammatory protein	1	1	1	40	40			
**Non-malarial antiparasitic**	**1**	**1**	**1**	**20**	**20**			
Suramin sodium	1	1	1	20	20			
**Protease Inhibitor**	**1**	**1**	**1**	**20**	**20**			
Ulinastatin	1	1	1	20	20			
**Case-Cotrol Studies**								
**ALL**	**2**	**3**						
**Angiotensin receptor modulator**	**1**	**1**						
ACE inhibitor	1	1						
**Monoclonal antibodies**	**1**	**2**						
Siltuximab	1	2						
**Cohort / Non-randomised** **Studies**								
**ALL**	**57**	**78**	**62**	**3330**	**25**	**10 - 59**	**4 - 500**	
**ATMP**	**16**	**19**	**17**	**634**	**10**	**10 - 30**	**5 - 300**	
Mesenchymal Stem Cells	5	6	6	150	17	10 - 30	10 - 66	
Covalescent plasma treatment	4	4	4	99	20	10 - 39.5	10 - 49	
Immunoglobulin from cured patients	2	3	1	5	5			
CAStem cells	1	1	1	20	20			
Umbilical Cord Mesenchymal Stem Cells	1	1	1	10	10			
Umbilical cord Wharton Jelly derived mesenchymal stem cells	1	1	1	5	5			
Interferon α1b	1	1	1	300	300			
gamma-Globulin	1	1	1	5	5	5		
Mechanical preventive anticoagulation	1	1	1	40	40			
**Antibiotic**	**1**	**1**						**1**
Ceftriaxone	1	1						1
**Anticoagulant**	**1**	**1**	**1**	**80**	**80**			
Heparin	1	1	1	80	80			
**Antifungal**	**1**	**1**	**1**	**80**	**80**			
Prophylactic antifungal therapy	1	1	1	130	130			
**Antimalarial**	**9**	**11**	**10**	**397**	**38**	**14 - 59**	**10 - 100**	**4**
Chloroquine	4	6	6	252	37	10 - 59	10 - 100	1
Hydroxychloroquine	5	5	4	145	38	22.5 - 50	20 - 50	3
**Antioxidant**	**1**	**1**	**1**	**500**	**500**			
Vitamin C	1	1	1	500	500			
**Antiviral**	**21**	**30**	**25**	**914**	**20**	**10 - 50**	**4 - 196**	**19**
Ritonavir	13	18	16	740	46	17 - 59.5	10 - 196	18
Lopinavir	10	15	13	709	50	20 - 60	14 - 196	15
Interferons	6	9	7	348	20	10 - 60	10 - 196	6
Azvudine	3	3	3	80	30	10 - 40	10 - 40	
Danoprevir	3	3	3	31	10	10 - 11	10 - 11	3
Oseltamivir	2	2	2	70	35	20 - 50	20 - 50	2
Tenofovir	1	1	1	60	60			1
LL-37 antiviral peptide	1	1	1	10	10			
Umifenovir	1	1	1	196	196			1
Favipiravir	1	1	1	50	50			
Azatanavir	1	1	1	50	50			1
Novaferon	1	1	1	10	10			
Fludase	1	1	1	4	4			
Emtricitabine	1	1	1	60	60			1
Remdesivir	1	1						
**Corticosteroids**	**3**	**3**	**3**	**122**	**50**	**20 - 52**	**20 - 52**	**1**
Corticosteroid_not specified	2	2	2	72	36	20 - 52	20 - 52	1
Glucocorticoid	1	1	1	50	50			
**Immunostimulatory**	**1**	**1**						**1**
Thymosin	1	1						1
**Immunosuppressive**	**1**	**1**						
Fingolimod	1	1						
**Monoclonal antibodies**	**7**	**7**	**4**	**500**	**40**	**20 - 230**	**20 - 400**	**2**
Tocilizumab	3	3	2	460	230	60 - 400	60 - 400	
Bevacizumab	1	1	1	20	20			
Eculizumab	1	1						1
Sarilumab	1	1						
Mepolizumab	1	1	1	20	20			1
**Non-specific anti-inflammatory** **or immunosuppressive**	**4**	**5**	**1**	**16**	**16**			**1**
Baricitinib	2	2						1
Escin	1	1						
Jacketinib hydrochloride	1	1	1	16	16			
Sodium Escinate	1	1						
Pulmonary arterial hypertension agent	1	1	1	10	10			
Sildenafil citrate	1	1	1	10	10			
**Sigma receptor modulator**	**1**	**1**						
Noscapine	1	1						
**Unable to classify**	**2**	**3**	**3**	**226**	**48**	**48 - 130**	**48 - 130**	
drug intervention	1	2	2	96	48	48 - 48	48 - 48	
diagnostic antifungal therapy	1	1	1	130	130			
**Quasi-randomised Stuides**								
**ALL**	**2**	**2**	**2**	**204**	**102**	**54 - 150**	**54 - 150**	
**ATMP**	**1**	**1**	**1**	**150**	**150**			
Micro-ecological preparation	1	1	1	150	150			
**Antimalarial**	**1**	**1**	**1**	**54**	**54**			
Hydroxychloroquine	1	1	1	54	54			
**Randomised Controlled Trials**								
**ALL**	**178**	**293**	**173**	**38445**	**50**	**30 - 90**	**10 - 10000**	
**ATMP**	**29**	**38**	**29**	**1072**	**30**	**16 - 50**	**10 - 100**	**2**
NK Cells	3	7	1	10	10	10 - 10	10 - 10	
Umbilical Cord Mesenchymal Stem Cells	6	7	6	151	30	16 - 30	15 - 30	1
Covalescent plasma treatment	7	7	7	345	50	25 - 75	15 - 100	
Mesenchymal Stem Cells	5	5	5	178	35	13 - 60	10 - 60	1
Standard Plasma	3	3	3	155	50	30 - 75	30 - 75	
Human menstrual blood-derived stem cells	1	2	2	28	14	10 - 18	10 - 18	
Umbilical cord blood mononuclear cells	1	1	1	15	15			
Immunoglobulin from cured patients	1	1						
Cell exosomes	1	1	1	30	30			1
rhG-CSF	1	1	1	100	100			
Interleukin-2	1	1	1	40	40			
Inactivated Mycobacterium vaccine	1	1	1	30	30			
Umbilical cord Wharton Jelly derived mesenchymal stem cells	1	1	1	20	20			
Stem Cell Educator	1	1						
**Angiotensin receptor modulator**	**2**	**2**	**2**	**390**	**195**	**100 - 290**	**100 - 290**	
Losartan	2	2	2	390	195	100 - 290	100 - 290	
**Anti-gout**	**2**	**2**	**1**	**3000**	**3000**			
Colchicine	2	2	1	3000	3000			
**Antiallergic**	**2**	**2**	**2**	**80**	**40**	**30 - 50**	**30 - 50**	**1**
Tranilast	1	1	1	30	30			
Ebastine	1	1	1	50	50			1
**Antibiotic**	**5**	**5**	**1**	**260**	**260**			**4**
Azithromycin	4	4		0				4
Carriomycin	1	1	1	260	260			
Anticoagulant	2	2	2	60	30	30 - 30	30 - 30	
Enoxaparin Sodium	2	2	2	60	30	30 - 30	30 - 30	
**Antifungal**	**1**	**1**						
Itraconazole	1	1						
**Antimalarial**	**44**	**64**	**29**	**24765**	**56**	**50 - 180**	**40 - 10000**	**19**
Hydroxychloroquine	33	46	15	13549	100	50 - 558	40 - 10000	15
Chloroquine	17	19	14	11216	50	40 - 150	40 - 10000	5
**Antioxidant**	**6**	**6**	**4**	**601**	**116**	**32 - 268.5**	**30 - 340**	**1**
Vitamin C	4	4	2	370	185	30 - 340	30 - 340	1
Alpha-lipoic acid	2	2	2	231	116	34 - 197	34 - 197	
**Antiviral**	**70**	**134**	**75**	**6950**	**50**	**30 - 116**	**10 - 620**	**74**
Ritonavir	33	56	29	2905	42	30 - 80	10 - 620	54
Lopinavir	32	50	28	3130	50	30 - 82	10 - 620	49
Interferons	24	36	20	2142	63	36 - 134.5	10 - 620	27
Favipiravir	11	19	15	655	30	20 - 50	10 - 120	6
Umifenovir	13	18	9	1005	80	50 - 190	15 - 260	7
Remdesivir	10	15	2	906	453	286 - 620	286 - 620	
Oseltamivir	3	9						7
Darunavir	3	5	1	40	40			5
Ribavirin	3	5	5	186	36	15 - 36	15 - 84	5
Novaferon	2	4	4	300	55	30 - 120	30 - 160	1
ASC09	3	3	1	80	80			3
Baloxavir marboxil	2	2	2	20	10	10 - 10	10 - 10	
Sofosbuvir	2	2	1	35	35			2
Cobicistat	1	1						1
Daclastavir	1	1	1	35	35			1
Danoprevir	1	1	1	30	30			1
Triazavirin	1	1	1	120	120			
Azvudine	1	1	1	10	10			
Ledispavir	1	1						1
**Corticosteroids**	**8**	**11**	**4**	**224**	**63**	**37 - 75**	**24 - 75**	**2**
Corticosteroid_not specified	7	10	4	224	63	37 - 75	24 - 75	2
Dexamethasone	1	1						
**Herbal Syrup**	**1**	**1**	**1**	**75**	**75**			**1**
Corostop	1	1	1	75	75			1
Coroguard	1	1	1	75	75			1
**Immunostimulatory**	**5**	**8**	**5**	**160**	**40**	**20 - 40**	**20 - 40**	**2**
Thymosin	3	5	4	140	40	30 - 40	20 - 40	2
Leukine	1	2						
Polyinosinic-polycytidylic acid	1	1	1	20	20			
**Immunosuppressive**	**2**	**2**						**1**
Thalidomide	2	2						1
**Kinase inhibitor**	**1**	**1**	**1**	**35**	**35**			**1**
Ruxolitinib	1	1	1	35	35			1
**Monoclonal antibodies**	**16**	**22**	**10**	**472**	**30**	**30 - 90**	**18 - 94**	**3**
Tocilizumab	6	9	5	334	90	30 - 90	30 - 94	2
Sarilumab	5	6						
Tozumab	1	1	1	30	30			1
Bevacizumab	1	1						
PD-1 blocking antibody	1	1						
Emapalumab	1	1	1	18	18			
Adalimumab	1	1	1	30	30			
Adamumab(Qletli)	1	1	1	30	30			1
Camrelizumab	1	1	1	40	40			
Ixekizumab	1	1	1	20	20			
**Mucolytic agent**	**3**	**4**	**3**	**166**	**68**	**30 - 68**	**30 - 68**	**1**
Acetylcysteine	2	3	3	166	68	30 - 68	30 - 68	
Bromhexine hydrochloride	1	1						1
**NSAIDs**	**1**	**1**						
Nonsteroidal anti-inflammatory drug	1	1						
**Non-specific anti-inflammatory** **or immunosuppressive**	**9**	**10**	**9**	**390**	**20**	**18 - 30**	**10 - 147**	**1**
Pirfenidone	3	3	3	187	20	20 - 147	20 - 147	
Sodium Escinate	1	2	2	40	20	10 - 30	10 - 30	
Anakinra	2	2	1	18	18			
Diammonium glycyrrhizinate	1	1	1	30	30			1
Macrophages suppression therapy	1	1	1	15	15			
Leflunomile	1	1	1	100	100			
**Protease Inhibitor**	**1**	**1**	**1**	**50**	**50**			
Ulinastatin	1	1	1	50	50			
**Unable to classify**	**5**	**5**	**4**	**430**	**95**	**15 - 200**	**10 - 230**	
Hydrogen peroxide	1	1	1	20	20			
Bismuth potassium citrate	1	1	1	170	170			
Dipyridamole	1	1	1	230	230			
Aviptadil	1	1		0				
Hormone	1	1	1	10	10			

Table includes 230 therapeutic studies with 354 study arms and 13 prevention studies with 26 arms. Four interventions with inhaled gases and one palliative care intervention (Dexmedetomidine) are excluded.

**Figure 4.**
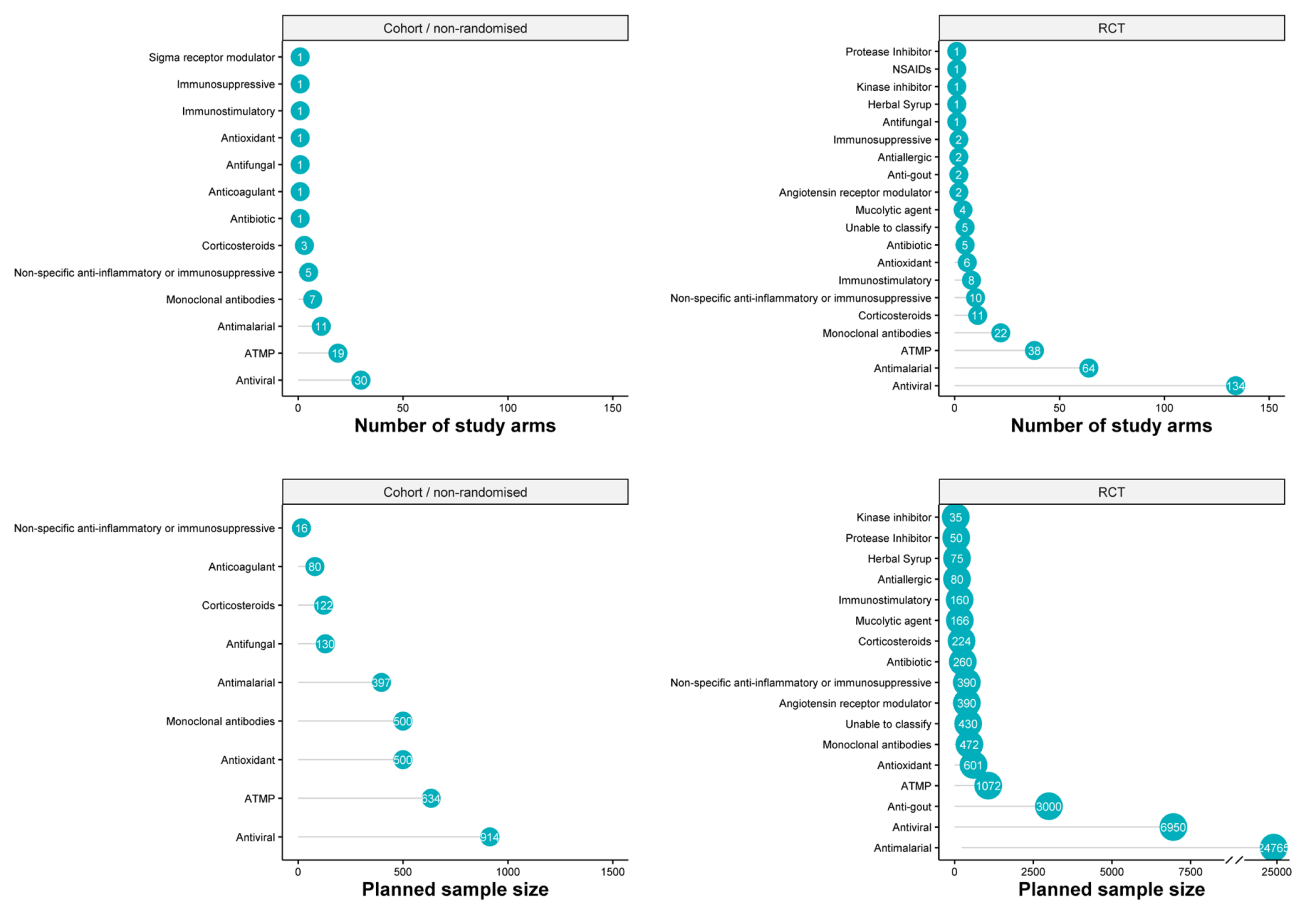
Number of study arms and planned sample sizes in cohort/non-randomised, and randomised studies. The number of study arms assessing each of the drug interventions is presented in upper panels. Planned sample size is presented in lower panels. The x-axis ranges are different for bottom panels with discontinuous x-axis at 8,000 for the bottom right panel. The number inside the dots present the number of arms (upper panels) and planned sample size (lower panels). Abbreviations: ATMP = Advanced Therapy Medical Products; RCT = Randomised controlled trial. The latest and previous versions of this figure are available as extended data ^13^.

Number of patients planned to be enrolled were available for 63% of study arms (244/385) with median (IQR, Range) of 40 (20–80, 4 – 10000) participants with highest sample sizes planned in RCTs. Irrespective of study design the most commonly reported intervention was antivirals, while the largest sample size was planned for antimalarials ( Figure 4).

### Pharmacological and ATMP Interventions assessed in RCTs

Of the 178 randomised control trials, there were a total of 293 arms assessing 80 pharmacological and ATMP interventions ( Table 1). Among RCTs, the most commonly used drug categories were antivirals (134 arms in 70 trials), antimalarials (64 arms in 44 trials), ATMP (Advanced Therapy Medicinal Products) (38 arms in 29 trials) and monoclonal antibodies (22 arms in 16 trials). Among antivirals assessed in RCTs, ritonavir featured in the most arms (56 arms in 33 trials with an overall planned sample size of at least 2,905 patients) followed by lopinavir (50 arms in 32 trials with an overall planned sample size of at least 3,130 patients) and interferons (36 arms in 24 trials, with an overall planned sample size of at least 2,142 patients). The two antimalarials assessed were hydroxychloroquine (46 arms in 33 trials, planned sample size of 13,549 patients) and chloroquine (19 arms in 17 trials, planned sample size of 11,216 patients). Of the ATMPs assessed in RCTs, the most frequently assessed were convalescent plasma treatment (7 arms in 7 trials, planned sample size of 345), umbilical cord mesenchymal stem cells (7 arms in 6 trials, planned sample size of 151), and mesenchymal stem cells (5 arms in 5 trials, planned sample size of 178). In total, 83 treatment arms in 52 studies included 38 different combinations of pharmacological or ATMP interventions ( Table 2). The most frequent combination of treatments to appear in RCT study arms was lopinavir/ritonavir (20 arms in 19 trials, planned sample size of at least 1,618 patients) alone; or combined with interferons (12 arms in 7 trials, planned sample size of at least 826 patients) or Hydroxychloroquine (4 arms in 4 trials, unknown sample size); or Hydroxychloroquine/Azithromycin (3 arms in 3 trials, unknown sample size). All other drug combinations were studied only in 2 (10 combinations, 26%) or 1 (24 combinations, 63%) study arm(s).

**Table 2.** Summary of drug combinations studied in case series, case-control, cohort/non-randomised, quasi-randomised and randomised studies. The latest and previous versions of this table are available as extended data ^13^.

			Number of arms	Sample Size			
Drug Name	Number of studies	Number of arms	with sample size	Total	Median	IQR	Range
**Cohort / Non-randomised Studies**							
**Antivirals**	**9**	**13**					
Lopinavir/Ritonavir	4	6	5	173	20	20 - 59	14 - 60
Danoprevir/Ritonavir	2	2	2	20	10	10 - 10	10 - 10
Interferons/Lopinavir/Ritonavir	2	2	2	101	51	41 - 60	41 - 60
Danoprevir/Interferons/Ritonavir	1	1	1	11	11		
Emtricitabine/Lopinavir/Ritonavir/Tenofovir	1	1	1	60	60		
Interferons/Lopinavir/Ritonavir/Umifenovir	1	1	1	196	196		
**Antiviral/Antimalarial**	**4**	**4**					
Azatanavir/Lopinavir/Ritonavir/Hydroxychloroquine	1	1	1	50	50		
Interferons/Lopinavir/Oseltamivir/Hydroxychloroquine	1	1	1	20	20		
Lopinavir/Oseltamivir/Ritonavir/Hydroxychloroquine	1	1	1	50	50		
Lopinavir/Ritonavir/Chloroquine	1	1	1	59	59		
**Antiviral/Immunostimulatory**	**1**	**1**					
Interferons/Thymosin	1	1					
**Antiviral/Non-specific anti-inflammatory or** **immunosuppressive**	**1**	**1**					
Lopinavir/Ritonavir/Baricitinib	1	1					
**Monoclonal antibodies/Antibiotic**	**1**	**11**					
Ceftriaxone/Eculizumab	1	1					
Mepolizumap/Corticosteroid_not specified	1	1	1	20	20		
**Randomised Controlled Trials**							
**Antivirals**	**34**	**53**					
Lopinavir/Ritonavir	19	20	14	1618	50	30 - 80	10 - 620
Interferons/Lopinavir/Ritonavir	7	12	6	826	56	10 - 75	10 - 620
ASC09/Ritonavir	2	2	1	80	80		
Interferons/Lopinavir	2	2	2	282	141	50 - 232	50 - 232
Interferons/Lopinavir/Ribavirin/Ritonavir	2	2	2	120	60	36 - 84	36 - 84
Interferons/Ribavirin	2	2	2	51	26	15 - 36	15 - 36
Interferons/Umifenovir	2	2					
Lopinavir/Oseltamivir/Ritonavir	1	2					
ASC09/Oseltamivir	1	1					
Cobicistat/Darunavir	1	1					
Daclastavir/Sofosbuvir	1	1	1	35			
Danoprevir/Ritonavir	1	1	1	30			
Favipiravir/Lopinavir/Ritonavir	1	1					
Interferons/Ritonavir	1	1	1	116			
Lopinavir/Novaferon/Ritonavir	1	1	1	30			
Oseltamivir/Ritonavir	1	1					
Ribavirin/Umifenovir	1	1	1	15			
**Antiviral/Antimalarial**	**7**	**13**					
Lopinavir/Ritonavir/Hydroxychloroquine	4	4					
Darunavir/Oseltamivir/Ritonavir/Hydroxychloroquine	1	2					
Favipiravir/Chloroquine	2	2	2	100	50	50 - 50	50 - 50
Interferons/Lopinavir/Ritonavir/Hydroxychloroquine	2	2					
Darunavir/Favipiravir/Ritonavir/Hydroxychloroquine	1	1					
Ledispavir/Lopinavir/Ritonavir/Hydroxychloroquine	1	1					
Oseltamivir/Hydroxychloroquine	1	1					
**Antiviral/Immunostimulatory**	**1**	**2**					
Darunavir/Thymosin	1	1	1	40	40		
Lopinavir/Ritonavir/Thymosin	1	1	1	40	40		
**Antiviral/Monoclonal antibodies**	**2**	**2**					
**Favipiravir/Tocilizumab**	**2**	**2**	**2**	**180**	**90**	**90 - 90**	**90 - 90**
**Antiviral/Antiallergic**	**1**	**1**					
Ebastine/Interferons/Lopinavir	1	1	1	50	50		
**Antiviral/Corticosteroids**	1	1					
Interferons/Umifenovir/Corticosteroid_not specified	1	1					
**Antiviral/Mucolytic agent**	**1**	**1**					
Interferons/Umifenovir/Bromhexine hydrochloride	1	1					
**Antiviral/Corticosteroids/Immunosuppressive**	**1**	**1**					
Interferons/Umifenovir/Corticosteroid_ns/Thalidomide	1	1					
**Antimalarial/Antibiotic**	1	4					
Hydroxychloroquine/Azithromycin	3	3					
Chloroquine/Azithromycin	1	1					
**ATMPs**	**4**	**1**					
Cell exosomes/Umbilical Cord Mesenchymal Stem Cells	1	1	1	30	30		
**ATMP/Kinase inhibitor**	**1**	**1**					
Mesenchymal Stem Cells/Ruxolitinib	1	1	1	35	35		
**Monoclonal antibodies**	**1**	**1**					
Adamumab(Qletli)/Tozumab	1	1	1	30	30		
**Antioxidant/Non-specific anti-inflammatory or** **immunosuppressive**	**1**	**1**					
Vitamin C/Diammonium glycyrrhizinate	1	1	1	30	30		
**Herbal Syrup**	**1**	**1**					
Coroguard/Corostop	1	1	1	75	75		

Table includes 66 therapeutic studies with 102 study arms and 2 prevention studies with 2 arms. No combinations were reported in case series, case-control or quasi-randomised studies.

### Reducing the fragmentation of results via graph-based data structures

Robust extraction of the results described above was hindered by the incompatible and inconsistent data fields, categorisation, and naming conventions across the many source trial registries. This lack of harmonisation limits the fidelity of results pooled across these sources. To overcome this and ensure reliable results, we implemented manual review of source registry records, further data extraction, and data harmonisation. This process of harmonisation has further enabled mapping of the results to a graph-based data structure that links data across key variables.

**Figure 5.**
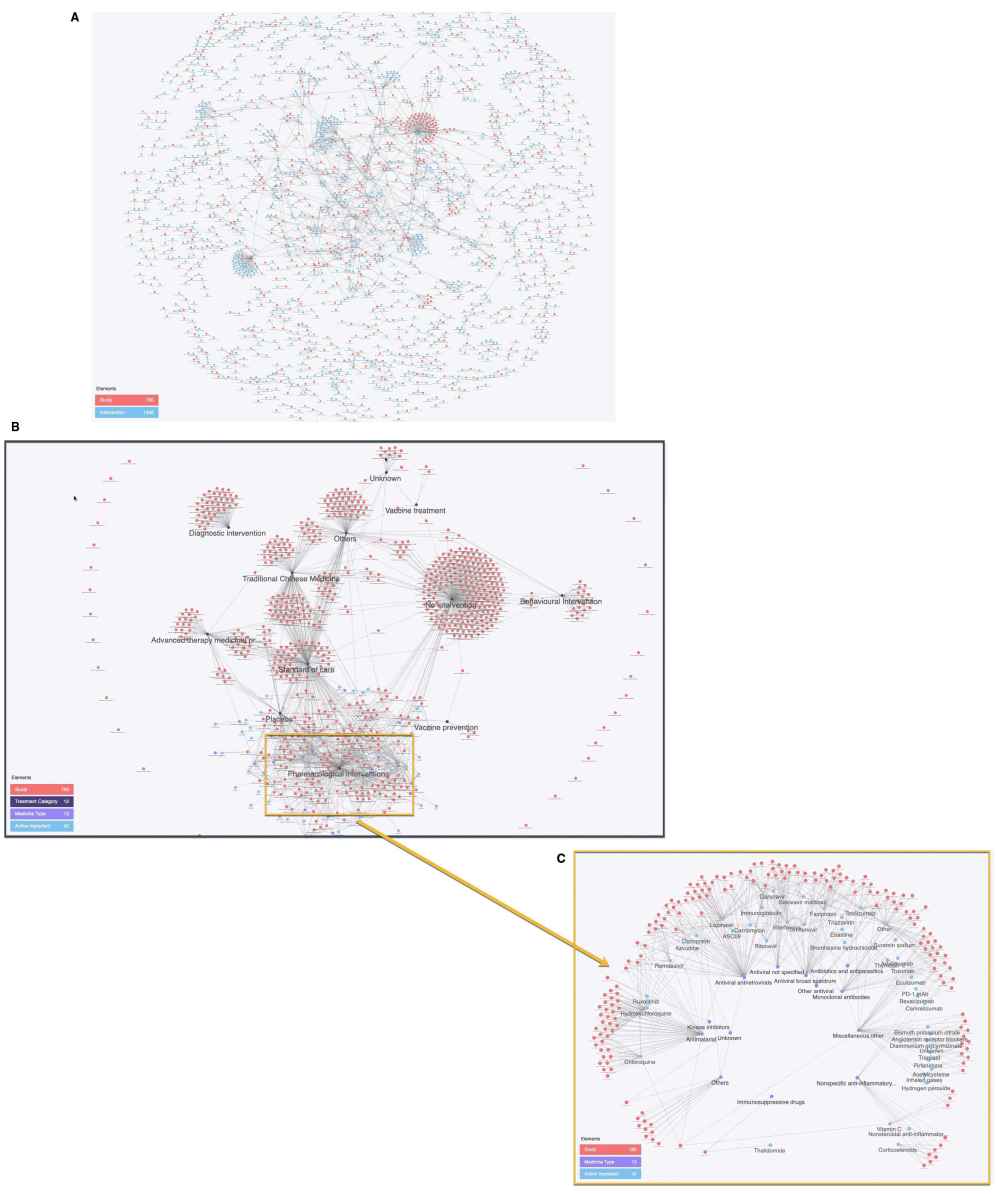
( **A**) Cognitive City showing graph network of intervention and trial from raw WHO ICTRP data. The network is highly disconnected due to the high number of redundant interventions that result from an uncontrolled vocabulary. ( **B**) Cognitive City showing graph network of intervention and trial using standardised IDDO COVID living systematic review data and ( **C**) Cognitive City sub-graph of 5b with intervention and trial using standardised data, for any trial with at least 1 pharmacological intervention arm. The network is highly connected due to the controlled intervention vocabulary and ontology. The latest and previous versions of this figure are available as extended data ^13^.

Data have been linked to support structured comparison of data, making results easier to visualise and interpret. Using the example of drug naming conventions, Figure 5a shows the highly fragmented outputs of raw drug names extracted from WHO ICTRP, compared to the harmonised network enabled by the standardised categorisation of the IDDO COVID systematic review data in Figures 5b and 5c.

Multiple interactive dashboards are built on top of the graph database to provide a simple interface to query the repository of clinical study data. More advanced interactive network visualizations allow for ad-hoc exploration of the underlying data. Data can be searched and filtered to exploit patterns based on the open meta-data generated by the systematic review, including type of interventions, treatment arms, study design, sample size, country, registry source, and strength of evidence, to find relevant trials that fit their specific criteria. The data and interactive platform are available at https://iddo.cognitive.city/ ^21^.

## Discussion

The COVID-19 clinical trial landscape is highly dynamic, changing on a daily basis. We present a comprehensive review of all registered trials up until 3 April 2020. The results highlight an unprecedented amount of research planned in a remarkably short period of time. While the mobilisation of the scientific community is admirable, this review illustrates a lack of coordination and duplication of effort that will ultimately delay definitive results of key interventions. The expected scientific value of many studies is difficult to gauge, however, limitations of many trial designs are apparent, for instance lack of controlled comparators, and observational studies and trials with inadequate sample size. The proliferation of such studies will likely result in underpowered studies individually unable to generate meaningful evidence for policy makers.

Planned numbers of patients to be enrolled in treatment arms were small across the majority of studies, even in RCTs (median = 134; 25 ^th^, 75 ^th^ percentile = 70, 300). It is difficult to comment on any individual studies in the review as they varied with respect to objectives and patient populations but small trials may not achieve sufficient power to detect clinically relevant treatment effects. For example, detection of 25% reduction in mortality with 80% power would require 2005, 906, 540, 356 or 247 patients in each arm assuming 10, 20, 30, 40, 50% mortality rate in the control arm, respectively. Similarly large sample sizes are needed for the assessment of improvement on the 7-level COVID Ordinal Outcomes Scale. It has been estimated that, 1128, 342, 160, 117 patients per arm are needed to ensure 80% power to detect an odds ratio of 1.25, 1.5, 1.75, 2.0 for improvement on the outcome on COVID Ordinal Outcomes Scale by day 15 ^23^. These estimates were derived based on the distribution of outcomes in the population studied and will not apply to all settings but sample sizes of similar magnitude may be expected. A significant reduction in the sample size needed may be achieved through the adaptive approach to study design ^24^, appropriate covariate adjustment ^25, 26^ and the use of an ordinal rather than binary outcome ^27^ and a number of studies with such designs have been recently planned/started.

While many studies are testing pharmacological treatment (n=204/728), a large number of trials (n=132/728) are testing traditional medicines, especially in China. A limited number of studies are testing diagnostic tools or are observing social parameters affected by the pandemic. Expectedly at this early stage of the research, very few vaccine trials were reported. The vast majority of studies are planning to recruit participants from high income and upper middle income countries, with only four studies planning to recruit participants from lower middle income and low middle income countries. This observed imbalance may reflect both challenges in funding mobilisation and the epidemiological pattern of the diseases of these regions. However, interpretation of this finding is nuanced as the number of trials from resource limited settings has increased over recent weeks. Similarly, focus areas and interventions for research are expected to change as research priorities evolve. This comprehensive analysis of data reported in trial registrations could help to identify research gaps and duplication, as well as guide funding priorities.

As with research on other novel interventions, vulnerable populations such as pregnant women, children or patients with co-morbidities are often excluded from trials and thus are under or unrepresented. Only nine studies indicated that pregnant women were eligible for inclusion. Reliable assessment of the inclusion of vulnerable populations among registered studies was hampered by the limited information included in study registries. The eligibility of pregnant women was unknown in roughly half of studies and the eligibility of the other at-risk groups was unable to be ascertained from the study registry in over 75% of registered studies.

### Medical product quality

These data provide vital evidence for understanding the rapidly changing landscape for what efficacious medical products will hopefully soon be available for the management of COVID-19 infection. As such evidence accrues, without global planning for production, distribution logistics, post-market surveillance, and actively detecting and acting on the inevitable substandard and falsified products, we risk a global crisis in access to and quality of anti-COVID medicines. Up to 7.8 billion people will be in dire need of a limited supply ^28^. There has already been a deluge of public health problems from quackery to substandard and falsified products, such as chloroquine, masks, diagnostic tests, ‘vaccines’ and hand sanitiser (see IDDO Medicine Quality Monitoring Globe) ^28, 29^. A ‘living’ system will help identify the most promising medical product interventions to prompt crucial pro-active planning for coordinated production and logistics for equitable access, optimal and appropriate prevent, detect and respond monitoring of product quality ^30^ and trials of portable devices for empowering post-market surveillance ^31^.

### Limitations and challenges

Although two individuals (at least one of whom was a medical doctor) reviewed each included trial record, extraction of manual variables was not blinded. Any changes made during extraction to the reported values such as study design categorisation were made on review of the totality of information provided within the entire registry record. While the rationale of the WHO International Standards for Clinical Trial Registries is undisputed ^32^, our review highlights substantial heterogeneity, inconsistencies and perceived errors in the recorded information across source country and regional registries. For example, as different trial registries have different classification systems for study design, there were 76 unique descriptions of study design as collected by the WHO ICTRP, with 66 studies containing no information. To allow a common classification scheme for comparisons across registries, each study was manually reattributed to one of 11 study design categories. Inevitably there is a degree of subjectivity in this process leading to possible errors in classification within our database; however, this was minimised by ensuring that all categorical attributes were clearly and prospectively standardised, defined and documented prior to extraction. Other explanations for observed inconsistencies in the original reported registrations include language barriers preventing accurate completion of study registrations in English, human error inherent in urgent registration during the pandemic, poor understanding of the terminology, and intentionally vague or poor reporting of information. While the current WHO International Standards for Clinical Trial Registries outlines minimum reporting guidance of what should be collected in registration records, consensus on how these details are collected limits the current utility of this information source alone. For this reason, caution should be raised in the interpretation of many initiatives currently presenting the raw WHO ICTRP registry data. Without harmonisation of data fields and common taxonomies and definitions, records across different source registries do not align.

### Sustainability and future work

As a living systematic review, weekly search updates are being conducted and have already identified more than 2,000 additional clinical trials registered since the 3 April 2020, that are being conducted in the context of COVID-19 (as at 26 May 2020). Cancellations also add to the constant flux in the number of registrations, with almost 10% of registered studies being cancelled before commencement; the majority of these occurred in China where a decline of COVID-19 cases has resulted in few patients eligible for enrolment. This further emphasises the need for collaboration particularly within countries and regions as many isolated trials initiated will similarly encounter a lack of patients available for enrolment as the outbreak’s infectivity changes over time. The WHO ICTRP was used as the primary source for this baseline review and while 3 source registries were also formally searched manually, some relevant study records from other registries published prior to our search cut-off date were not yet accounted for in the available WHO ICTRP export. As an example, while analysing the baseline results we were made aware of an additional 107 clinical trials in the Iranian Registry of Clinical Trials (IRCT) that were not reflected in WHO ICTRP and are therefore not included in this analysis ^33, 34^. Almost six months after COVID-19 was identified, the expansion of clinical research is far from slowing. Hence a living systematic review method is appropriate and sustained updates will be necessary for the foreseeable future. Ongoing search updates are scheduled to be incorporated into the open, online and searchable IDDO COVID-19 Cognitive City platform.

During completion of the first phase of this project, initiatives with parallel objectives were identified ^35^. Of the few online clinical trial visualisation tools that are similarly going back to source registries and manually reviewing records for extraction, there are noticeable differences in approach and agenda, but also synergies across some variables. Standardisation and harmonisation of these comparable variables, matched with an ethos of open sharing of extended data from all projects could help to minimise the duplication of work across research organisations. Therefore, future work is planned to assess these synergies across tools and assess the feasibility and willingness for coordination and collaboration moving forward. Such an interchange could only enhance the utility of our collective efforts.

Furthermore, additional work planned, as requested by collaborators from the COVID-19 Clinical Research Coalition, will include a categorisation of trials assessing affordable and readily available interventions for deployment in low resource settings that are feasibly adaptable to such health care systems. At a later stage, this living review will allow prioritisation of research targets for individual patient data meta-analysis and will support other COVID-19 Clinical Research Coalition efforts ^36^.

The continued work of this living systematic review will allow a more dependable overview of interventions tested, predict the strength of evidence likely to be generated, allow fast and informative filtering of relevant trials for specific user groups and provide the rapid guidance needed by investigators to avoid duplication of efforts.

## Data availability

### Underlying data

Harvard Dataverse: Associated data for: IDDO Living Systematic Review for COVID-19 Clinical Trial Registrations. https://doi.org/10.7910/DVN/YAZVZE ^13^

This project contains the following underlying data:

- Baseline_Supplementary_file_Screened_Included_ClinicalTrialRegistrations.xls (List of de-duplicated registrations screened, included and excluded from the systematic review of records)
- Baseline_underlying_data_missing drug names_extract_30APR2020.xls (Table providing drug name corrections required for Script_drug summary_7MAY2020.do file)
- Baseline_underlying_data_2020-05-07_iddo_lsr_covid-19_sr.tab (Underlying data of baseline results in .dta format)
- Baseline_underlying_data_2020-05-07_iddo_lsr_covid-19_sr-1.tab (Underlying data of baseline results in .csv format)

### Extended data

The completed PRISMA checklist, REDCap variable and data dictionaries and supplementary materials are available.

Harvard Dataverse: Associated data for: IDDO Living Systematic Review for COVID-19 Clinical Trial Registrations. https://doi.org/10.7910/DVN/YAZVZE ^13^

This project contains the following extended data:

- Baseline_Figure 1. PRISMA diagram.pdf (Baseline version of Figure 1, PRISMA flowchart)
- Baseline_Figure2.tif (Baseline results version of Figure 2)
- Baseline_Figure 3.tif (Baseline results version of Figure 3)
- Baseline_Figure_4.tiff (Baseline results version of Figure 4)
- Baseline_Figure 5a.tiff (Baseline results version of Figure 5a)
- Baseline_Figure 5b,c.tiff (Baseline results version of Figure 5b and c)
- Baseline_Table1.xls (Baseline results version of Table 1)
- Baseline_Table2.xls (Baseline results version of Table 2)
- Baseline_Supplementary Table 1.pdf (Supplementary table of eligible populations for inclusion)
- Baseline_Supplementary_file_IDDO COVID19_DB_VariableDictionary.pdf (Variable dictionary for data extraction of included records)R script for cross verification of study level summary.R (R script to generate a basic study level summary with unique number of studies by country and eligibility of risk populations)
- R script for generation of Figure 4.R (R script to generate Figure 4)
- Script_drug summary_7MAY2020.do (Stata do file to generate Baseline Table1.xls and Baseline Table2.xls)
- Script_extended_data_table1.do (Stata do file to generate Supplementary Table 1)
- Script_figure_2.do (Stata do file to generate figure 2)
- Script_figure_3a.do (Stata do file to generate figure 3 - bar chart)
- Script_figure_3a.do (Stata do file to generate figure 3 map)
- Script_prepare_data_for_figure_3b.do (R script to generate figure 3 map)
- Script_scheme-iddocovid.scheme (Stata graphic scheme file used)

### Reporting guidelines

Harvard Dataverse: PRISMA checklist and flow diagram for ‘Baseline results of a living systematic review for COVID-19 clinical trial registrations’ https://doi.org/10.7910/DVN/YAZVZE ^13^
