# Supplementary material for: Baseline results of a living systematic review for COVID-19 clinical trial registrations
Source: Wellcome Open Res. 2020 Jun 2;5:116. [Version 1] doi: 10.12688/wellcomeopenres.15933.1 (PMC7610178; doi:10.12688/wellcomeopenres.15933.1)
Supplement: Peer review history

# Peer review: Reviewer response for version 1

**DOI:** 10.21956/wellcomeopenres.17477.r40082

**Published:** 2020-10-15

**Authors:** Arnav Agarwal

**Affiliations:** 1 Department of Medicine, University of Toronto, Toronto, Canada

**Summary:**

 This is a living systematic review of clinical study registrations (clinical trials and otherwise) for the diagnosis, treatment and prevention of COVID-19, an important endeavour in the midst of the pandemic and a concurrent infodemic with rapid evidence proliferation. 

 The review found:

- Rapid proliferation of studies in a short period of time, as expected.
- Majority of studies had small sample sizes and single-centre study designs, and were likely underpowered for outcomes like mortality.
- Majority of studies focused on recruitment in China, and the majority focused on recruitment from institutions based in high income and upper middle-income countries.
- Significant focus on pharmacological treatments and traditional medicines, the latter being predominantly Chinese-based.
- Majority of pharmacological intervention studies evaluated antivirals, antimalarials, ATMPs and monoclonal antibodies.
- There were very few vaccine studies.
- There are few studies examining social parameters affecting the pandemic.

**Feedback:**

1. This is a methodologically sound review with a comprehensive search strategy, clear review process, and robust analysis. The choice of a living systematic review design is well-justified. 
2. The 'Cognitive CIty' visualizations are an excellent innovation. Congratulations.
3. Is there data on type of funding across studies?
4. Is there data on status of ongoing studies (i.e. planned, ongoing, etc.)? Is there data on planned completion? 
5. Perhaps article updates more frequently than every 6 months would be warranted, given the rapidly evolving state of the evidence.
6. Discussion around other existing databases with a similar focus on creating a living repository of clinical studies planned and underway may be added. One example is COVID-evidence.
7. Perhaps a call for more standardization and consistency across study registrations is appropriate, given the observation that there was significant variability in data available.
8. For eligibility criteria: consider explicitly specifying that you are interested in treatment studies for those with both suspected and confirmed SARS-CoV-2, primary and secondary prevention studies involving those without primary SARS-CoV-2 infection and those with a previous infection, and diagnosis studies across all populations.
9. For the 29 records excluded for not including COVID-19 patients, this seems to run counter to the eligibility criteria specified: "All clinical trial registrations either being planned, or currently underway to diagnose, treat or prevent COVID-19 were eligible for inclusion in the baseline results of this review. This included patients known to be infected with COVID-19 as well as healthy volunteers, healthcare workers or other patient populations where health related outcomes were assessed in the context of COVID-19". This is likely semantics, but great if this could be clarified.
10. This is not the main focus of the analysis, but it may be interesting to see the reasons for termination for the trials identified, and what interventions were being examined.
11. For 'Eligible populations', on what basis were the given comorbidities selected? They are certainly relevant, but wonder if these are the ones most commonly reported?
12. Great discussion re: many small studies likely being insufficiently powered for major outcomes of interest.
13. Great discussion re: vulnerable populations. I think rather than their being under-represented, patients with comorbidities are likely under-reported in study registrations.
14. Great discussion re: sustainability and future work.
15. There is a clear role for this living review in the evidence ecosystem along with other living evidence synthesis efforts around emerging evidence on treatment, prevention, diagnostics and prognostics. Perhaps mention regarding the role of this living review in the context of this broader evidence ecosystem would be helpful.
16. It may be interesting to track study registrations across time to see the evolution of which interventions are being studied in the context of constantly-emerging new evidence.
17. Consider standardizing use of 'clinical study' registrations throughout the study, including the title.
18. The manuscript is very well-written, and I have no other specific comments in this regard.

Are the rationale for, and objectives of, the Systematic Review clearly stated?

Yes

Is the statistical analysis and its interpretation appropriate?

Yes

Have the search and update schedule been clearly defined and justified?

Yes

Is the living method justified?

Yes

Are sufficient details of the methods and analysis provided to allow replication by others?

Yes

Are the conclusions drawn adequately supported by the results presented in the review?

Yes

Reviewer Expertise:

Methodological focus: systematic reviews, clinical guideline development. Clinical focus: general internal medicine.

I confirm that I have read this submission and believe that I have an appropriate level of expertise to confirm that it is of an acceptable scientific standard.

# Peer review: Reviewer response for version 1

**DOI:** 10.21956/wellcomeopenres.17477.r38934

**Published:** 2020-06-05

**Authors:** Julia C. Cutts

**Affiliations:** 1 Burnet Institute, Melbourne, Australia

This living systematic review represents an important resource to enable researchers, clinicians, and other stakeholders to gain synthesized, up-to-date information on COVID-19 clinical trial registrations. Importantly, a living systematic review approach is highly justified in this context given the frequency with which new COVID-19 clinical trials are currently being registered.

 Important findings gleaned from this baseline results study include the identification of relatively small proposed samples sizes for most of the registered studies, the relative dearth of studies to be conducted in low or lower middle-income countries, the insufficiency of information regarding eligibility of study participants, the large number of studies testing traditional medicines, and the limited number of studies testing diagnostic tools or examining social parameters of the pandemic. As well as tabulating and graphing key characteristics of the registered COVID-19 clinical trials, the authors present engaging “Cognitive City” visualizations of the registered trials by intervention.

 Conclusions drawn in the paper are supported by the results presented and the limitations and risks of bias are clearly addressed.

 The paper is scientifically sound, but I would recommend the following minor changes to improve its clarity:

 Whilst detailed in the review protocol, I would suggest that the information sources and search strategy is briefly summarized in this manuscript. For example, whilst it is inferred from the flow diagram and the text in the “Information sources and search strategy” section that ICTRP was the primary source of information, I would suggest including a more explicit statement to this effect.

 Table 1: In this table the data are stratified by study design, but the current text formatting makes it difficult to discern the individual study design sections.  For example, it is difficult to see where the data for “Case Series” ends and the data for “Cohort/Non-randomised Studies” begins. Perhaps reformat so that the study design headings are differentiated from the Drug names (e.g. using indenting or variation in bold or italics, as much as the journal’s prescribed table formatting will allow). Alternatively data for each study design could be presented in separate tables, but this may be too cumbersome.

 Table 1: Typographical errors to be corrected (Case-Cotrol Studies; Quasi-randomised Stuides)

 Medical product quality section within Discussion, page 18: The final sentence (below) needs to be amended. The gist of it is understandable, but it is grammatically flawed (see underline) and would perhaps be easier to digest if broken up into two sentences.

 “A ‘living’ system will help identify the most promising medical product interventions to prompt crucial pro-active planning for coordinated production and logistics for equitable access, optimal and appropriate prevent, detect and respond monitoring of product quality and trials of portable devices for empowering post-market surveillance.”

Are the rationale for, and objectives of, the Systematic Review clearly stated?

Yes

Is the statistical analysis and its interpretation appropriate?

Yes

Have the search and update schedule been clearly defined and justified?

Yes

Is the living method justified?

Yes

Are sufficient details of the methods and analysis provided to allow replication by others?

Yes

Are the conclusions drawn adequately supported by the results presented in the review?

Yes

Reviewer Expertise:

Infectious disease epidemiology, malaria immunology, sero-surveillance, systematic reviews, clinical trials.

I confirm that I have read this submission and believe that I have an appropriate level of expertise to confirm that it is of an acceptable scientific standard.
